# Biodistribution of a Mitochondrial Metabolic Tracer, [^18^F]F-AraG, in Healthy Volunteers

**DOI:** 10.1155/2022/3667417

**Published:** 2022-08-08

**Authors:** Jelena Levi, Heying Duan, Shahriar Yaghoubi, Juliet Packiasamy, Lyna Huynh, Tina Lam, Faiq Shaikh, Deepak Behera, Hong Song, Joseph Blecha, Salma Jivan, Youngho Seo, Henry F. VanBrocklin

**Affiliations:** ^1^CellSight Technologies Incorporated, San Francisco, California, USA; ^2^Department of Radiology, Stanford University, Palo Alto, California, USA; ^3^Department of Radiology and Biomedical Imaging, University of California San Francisco, San Francisco, California, USA

## Abstract

**Purpose:**

[^18^F]F-AraG is a radiolabeled nucleoside analog that shows relative specificity for activated T cells. The aim of this study was to investigate the biodistribution of [^18^F]F-AraG in healthy volunteers and assess the preliminary safety and radiation dosimetry.

**Methods:**

Six healthy subjects (three female and three male) between the ages of 24 and 60 participated in the study. Each subject received a bolus venous injection of [^18^F]F-AraG (dose range: 244.2–329.3 MBq) prior to four consecutive PET/MR whole-body scans. Blood samples were collected at regular intervals and vital signs monitored before and after tracer administration. Regions of interest were delineated for multiple organs, and the area under the time-activity curves was calculated for each organ and used to derive time-integrated activity coefficient (TIAC). TIACs were input for absorbed dose and effective dose calculations using OLINDA.

**Results:**

PET/MR examination was well tolerated, and no adverse effects to the administration of [^18^F]F-AraG were noted by the study participants. The biodistribution was generally reflective of the expression and activity profiles of the enzymes involved in [^18^F]F-AraG's cellular accumulation, mitochondrial kinase dGK, and SAMHD1. The highest uptake was observed in the kidneys and liver, while the brain, lung, bone marrow, and muscle showed low tracer uptake. The estimated effective dose for [^18^F]F-AraG was 0.0162 mSv/MBq (0.0167 mSv/MBq for females and 0.0157 mSv/MBq for males).

**Conclusion:**

Biodistribution of [^18^F]F-AraG in healthy volunteers was consistent with its association with mitochondrial metabolism. PET/MR [^18^F]F-AraG imaging was well tolerated, with a radiation dosimetry profile similar to other commonly used [^18^F]-labeled tracers. [^18^F]F-AraG's connection with mitochondrial biogenesis and favorable biodistribution characteristics make it an attractive tracer with a variety of potential applications.

## 1. Introduction

Intracellular nucleotide pools are intricately controlled as they critically affect cells' genomic stability, growth, proliferation, and survival [1]. The increased cellular metabolism and high demand for nucleotides in transformed, rapidly proliferating cancer cells, are the basis for the use of nucleoside analogs as chemotherapeutic agents. Using the nucleotide salvage pathway, nucleoside analogues, designed to mimic their naturally occurring counterparts, get phosphorylated by nucleoside kinases resulting in accumulation of triphosphorylated nucleotide analogues. The accumulated nucleotide analogues impair cancer cell growth by inhibiting enzymes essential for nucleic acid synthesis and leading to chain termination by being incorporated into DNA [2]. A range of pyrimidine and purine analogues is used in the treatment of hematological malignancies as well as solid tumors [3]. A deoxyguanosine analog, 9-*β*-D-arabinofuranosylguanine (AraG), was extensively investigated for its selective toxicity toward T leukemic cells, but poor solubility prevented its clinical use. Nelarabine, AraG's prodrug with improved solubility, is FDA-approved for treatment of patients with T cell acute lymphoblastic leukemia (ALL) and T cell lymphoblastic lymphoma [4]. AraG's T cell selectivity motivated the development of [^18^F]F-AraG as an imaging agent for activated T cells [5]. Administered in trace amounts, [^18^F]F-AraG, without toxicity, allows *in vivo* probing of T cell activity. Activated T cells play a key role in a range of processes that include both host-beneficial antitumor and antiviral immunity, and host-damaging immune response in graft vs. host disease (GvHD) and multiple sclerosis. Non-invasive tracking of activated T cells may allow assessment of proper or aberrant immune function and enable timely treatment interventions or modifications.

Mirroring AraG's behavior [6], [^18^F]F-AraG, enters T cells via nucleoside transporters and is trapped intracellularly through phosphorylation primarily by deoxyguanosine kinase (dGK) [7–9]. dGK is a rate-limiting mitochondrial kinase critical in supplying triphosphate nucleotides for mitochondrial DNA synthesis (mtDNA) [10]. Genetic dGK deficiency results in mtDNA depletion and devastating hepatocerebral syndrome [11]. The key to [^18^F]F-AraG's ability to visualize activated T cells lies in its association with mitochondrial biogenesis mediated through the action of mitochondrial dGK. Mitochondrial metabolism and biogenesis are tightly coupled to T cell function [12, 13]. In response to activation, T cells rapidly undergo metabolic reprogramming and dramatically increase both mitochondrial mass and mtDNA [14, 15]. Immunosuppressive tumor microenvironment affects T cell activation and effector function resulting in tumor infiltrating T cells with reduced mitochondrial function and mass [16]. T cell exhaustion, detrimental for antitumor immunity, is induced by the loss of mtDNA and is associated with altered nucleotide biosynthesis and mitochondrial dysfunction [17, 18].

In addition to dGK, mtDNA maintenance is strongly controlled by the activity of sterile alpha motif and HD-domain containing protein 1 (SAMHD1) [19]. SAMHD1, a key regulator of deoxyribonucleoside triphosphate (dNTP) pools and well-known for its HIV-1 restricting ability, dephosphorylates dNTPs, limiting the amount of building blocks available for DNA replication [20, 21]. Cells with an increased need for DNA synthesis, such as proliferating fibroblasts, transformed, or activated T cells, downregulate SAMHD1, allowing accumulation of dNTPs needed for nuclear and mtDNA synthesis. Low expression of SAMHD1 in T cell leukemia cells was found to be the critical determinant of AraG triphosphate's cellular accumulation and consequent sensitivity to nelarabine treatment [22]. The significantly lower expression of SAMHD1 mRNA in T-ALL cells than in any other tumor cell line in the Cancer Cell Line Encyclopedia database provides a clue into [^18^F]F-AraG's selectivity for T cells. Overall, the finely tuned mitochondrial biogenesis achieved through the interplay between SAMHD1 and dGK offers the basis for [^18^F]F-AraG's specificity for T cells [8, 9], a rare characteristic in metabolic tracers.

[^18^F]F-AraG has been evaluated in preclinical models of rheumatoid arthritis, GvHD, multiple sclerosis, and cancer [8, 9, 23–25]. Its utility in evaluating response to immunotherapies is currently investigated in multiple clinical trials. Here, we report radiation dosimetry data and discuss biodistribution in healthy subjects as it relates to signal specificity and mechanism of uptake.

## 2. Materials and Methods

### 2.1. [^18^F]F-AraG Synthesis

[^18^F]F-AraG was prepared by UCSF Radiochemistry facility according to the approved IND Chemistry Manufacturing and Control (CMC) procedures. A published procedure was modified for the use on Neptis PET synthesizer [5]. Briefly, [^18^F]fluoride (n.c.a.) in [^18^O]water was passed through a QMA-carbonate cartridge, eluted with 0.55 *μ*L of a phase transfer catalyst solution containing Kryptofix_222_ (6 mg) and K_2_CO_3_ (1.25 mg) in 1 : 1 acetonitrile/water. The K_222_/K[^18^F]F complex was heated and dried under nitrogen at 95°C for 3 min, followed by 3 × 100 *μ*L additions of acetonitrile and azeotropic evaporation under nitrogen flow and vacuum. After cooling to 60°C, a 1.2 mL solution containing [^18^F]F-AraG precursor (7-10 mg) in acetonitrile/2-methyl-2-butanol (1 : 5) is added to the reactor, and the solution was heated to 115°C for 30 min. After cooling down to 60°C, 1.2 mL of 0.5 M sodium methoxide was added to the reaction mixture and the solution heated at 100°C for 10 min. Reaction mixture was cooled to 60°C, 1.7 mL of 1 N HCl was added, and the solution was heated at 100°C for 10 min. The reaction mixture was cooled to 50°C and diluted with a solution of 1 mL of 1 N NaHCO3 and 2.5 mL water for injection. The solution was injected and purified on an HPLC Luna C18(2) semipreparative reversed-phase column (5 *μ*m, 10 × 250 mm). Desired peak was collected between 14 and 16 minutes and filtered on-line through a 0.22 *μ*m Millex GV sterile filter. The radiochemical yield was approximately 4% (decay corrected to End of Synthesis) with a synthesis time of 90 minutes. Approximately 0.6 mL of final product was removed aseptically for quality control tests.

### 2.2. Cell Studies

Human PBMCs were isolated from whole blood using lymphoprep density gradient solution (Stemcell Technologies). CD8^+^ T cells were purified from PBMCs using CD8 MicroBeads (Miltenyi Biotec). Antigen-presenting cells (APCs) were isolated from PBMCs by depleting CD4^+^ and CD8^+^ cells. APCs were pulsed with flu peptide (CEF1 influenza matrix protein M1 (58-66) (RP19978, GenScript)) at 15 nM for 8 hrs. Isolated CD8 T (negative isolation) cells were cocultured (5 : 1 ratio) with antigen-pulsed APCs for 72 h. CD8^+^ cells were subjected to tracer uptake and flow cytometry [9]. The follwoing antibodies were used for flowcytometry: CD3 Monoclonal Antibody (UCHT1), APC-eFluor 780, eBioscience (47-0038-420), V500 Mouse Anti-Human CD8, BD Biosciences (560774) SAMHD1 Recombinant Rabbit Monoclonal Antibody (JU56-04), Invitrogen (MA5-32810), Alexa Fluor® 647 Donkey anti-rabbit I (406414), FITC anti-human CD69 Antibody (310904), PE anti-human CD279 (PD-1) Antibody (367404).

### 2.3. Study Participants

All human subject studies were conducted under an UCSF IRB and radiation safety committee approved protocols. *Informed consent was obtained from all individual participants included in the study.* Additionally, clinical safety, pharmacokinetics, and dosimetry studies were conducted under Food and Drug Administration (FDA) exploratory investigational new drug number 123591 (clinical trial registration no. NCT02323893). Three male and three female healthy volunteers between the ages 24 and 60 years were enrolled. Pregnant and breast-feeding women were excluded from enrollment.

### 2.4. Image Acquisition and Processing

Following voluntary informed consent, each enrolled subject was prepared for [^18^F]F-AraG image acquisition and safety monitoring. Each enrolled subject received a bolus venous injection of [^18^F]F-AraG (dose range: 244.2–329.3 MBq), followed by four consecutive whole-body scans, covering the vertex to the midthigh on positron emission tomography/magnetic resonance imaging (SIGNA PET/MR, GE Healthcare). The duration of scan at each bed position was at minimum 180 seconds. Dixon MRI was acquired for MR-based attenuation correction implemented in PET reconstruction. During the PET imaging session blood samples were collected at 1, 3, 5, and 10 minutes after [^18^F]F-AraG injections and then at every 30 minutes interval up to 3 hours after [^18^F]F-AraG injections for blood time activity analysis.

Images were reviewed and analyzed by an experienced nuclear medicine physician (HD) using MIM software version 7.1.0 (MIM software, Cleveland, OH, USA). When feasible, a whole organ contour was drawn; otherwise, a region of interest (ROI) of 1 cm^3^ was laid upon the structure. For the blood pool, the same sized ROI was placed centrally in the lumen of the aortic arch. Mean standard uptake values (SUV) were collected for all structures for all four time points.

### 2.5. Biodistribution and Radiation Dosimetry

Reconstructed and attenuation corrected whole-body [^18^F]F-AraG images acquired at 4 consecutive time points starting upon injection of [^18^F]F-AraG were used to quantify ^18^F radioactivity present in various organs of each healthy subject. Activity was computed in the heart, liver, intestine, kidneys, lungs, bladder, brain, and spleen of each subject at all four time points, and the remainder of the body was computed by subtracting measured organ activities from the whole-body activity. The area under the time-activity curves were calculated for each organ and used to derive time-integrated activity coefficient (TIAC, also known as residence time). TIACs were then input for absorbed dose and effective dose calculations using OLINDA.

## 3. Results

### 3.1. Higher Accumulation of [^18^F]F-AraG in Antigen Stimulated T Cells Is Associated with Lower Levels of SAMHD1

**Figure 1:**
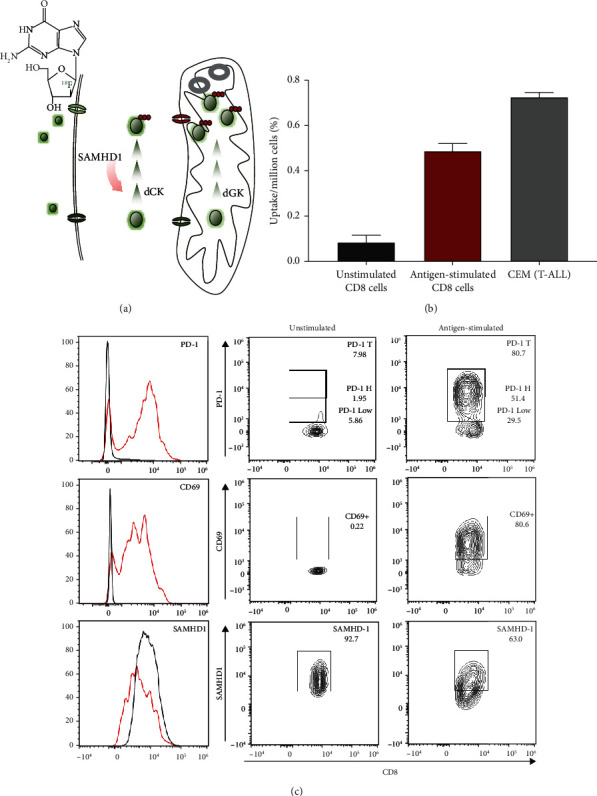
SAMHD1 and [^18^F]F-AraG uptake in antigen stimulated T cells. (a) The proposed mechanism of uptake. [^18^F]F-AraG is transported into cells via nucleoside transporters, followed by the rate-limiting phosphorylation by mitochondrial deoxyguanosine kinase (dGK). Once triphosphorylated, [^18^F]F-AraG can be incorporated into mtDNA or be exported from mitochondria where SAMHD1 can dephosphorylate triphosphate providing an opportunity for the unphosphorylated [^18^F]F-AraG to be exported from the cell. Overall, optimal trapping of [^18^F]F-AraG may be achieved in cells with high mitochondrial biogenesis and low expression of SAMHD1. (b) Accumulation of [^18^F]F-AraG in CEM, unstimulated and antigen-stimulated T cells. Antigen-stimulated T cells show 6-fold higher uptake than the unstimulated T cells. T-ALL CEM cells served as a positive control and showed the highest accumulation of tracer. (c) Antigen-stimulated CD8 cells (red line) show higher levels of PD-1 and CD69 and lower levels of SAMHD-1 than unstimulated cells (black line).

We had shown that immune cells express different levels of nucleoside transporters and dGK that affect [^18^F]F-AraG's uptake and retention [9]. To investigate the association between SAMHD1 levels and [^18^F]F-AraG's cellular accumulation, we determined the level of SAMHD1, activation markers PD-1 and CD69 and tracer uptake in CD8^+^ cells cocultured with flu peptide pulsed dendritic cells. Compared to naïve cells, antigen stimulated CD8^+^ cells had lower expression of SAMHD1, higher expression of PD-1 and CD69, and increased uptake of [^18^F]F-AraG (Figure 1).

### 3.2. Demographic Data and Imaging Protocol

Six subjects, three female and three male, were enrolled in the study. The mean age was 44.7 (range: 24–60 years), the mean weight 63.3 kg (range: 46.4–92.1 kg), and the mean administered dose 307.1 MBq (range; 244.2–329.3 MBq) (Table 1). Participants' ECG, blood pressure, blood count, and pulse oximetry were monitored. No clinically significant changes were found for any participant following [^18^F]F-AraG administration with follow-up at 24 hours and 1 week postinjection.

The whole-body scans were obtained at four time points post tracer injection, each scan lasting 30 minutes. The start time for scans 2 to 4 differed slightly between participants and is shown in Table 2.

### 3.3. Biodistribution

**Figure 2:**
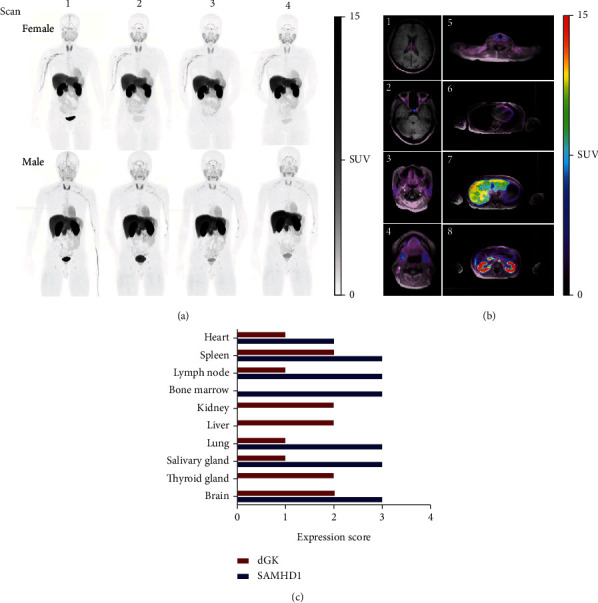
[^18^F]F-AraG PET images. (a) Whole body maximum intensity projection images at different time points in a representative female and male subject. (b) Axial slices of fused PET/MRI showing notable uptake in (1) choroid plexus, (2) pituitary gland, (3) parotid gland, (4) submandibular gland, (5) thyroid gland, (6) heart, (7) liver, and (8) kidneys. (c) Tissue expression of dGK and SAMHD1, extracted from the Human Protein Atlas; 0, 1, 2, and 3 signify no, low, medium, and high expression, respectively.

Maximum intensity projection (MIP) and whole-body distribution of [^18^F]F-AraG at different time points after tracer administration in a representative female and male participant are shown in Figure 2(a). The highest uptake was observed in the kidneys and liver, with prominent signal noted in the urinary bladder, choroid plexus, pituitary, submandibular, parotid, and thyroid glands, heart, and pancreas (Figure 2(b)). Low tracer uptake was observed in the brain, lung, bone marrow, and muscle.

**Figure 3:**
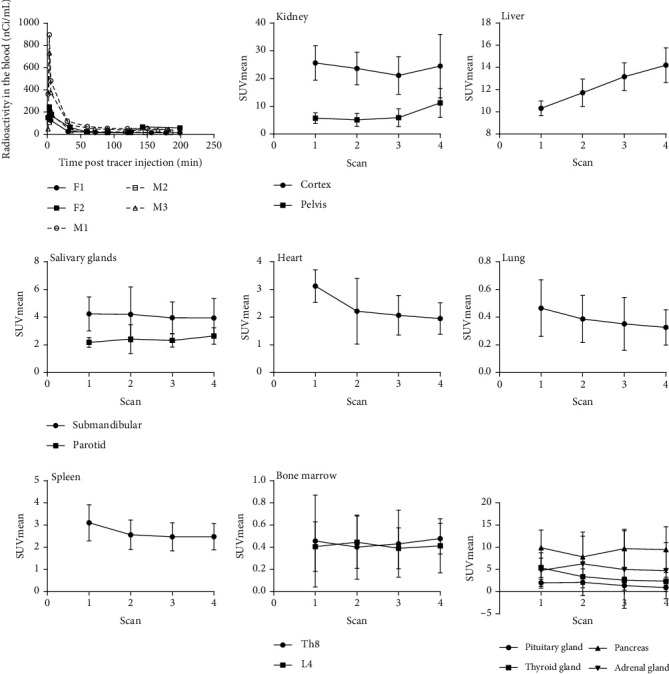
Time-activity curves for the blood and eight source organs in six study participants.

Radioactivity is rapidly cleared from the blood (Figure 3). While the signal in the heart, salivary glands, spleen, and bone marrow did not change appreciably post 2^nd^ scan (approximately 45 minutes posttracer injection), the signal in the liver continued to increase over time.

### 3.4. Radiation Dosimetry

The absorbed dose estimates for the six participants are listed in Table 3. The highest absorbed doses, 0.3063 and 0.2370 mGy/MBq for female and male subjects, respectively, were estimated for the kidneys and the second highest for the liver with 0.0721 and 0.0570 mGy/MBq for female and male subjects, respectively. The testes in male subjects and thyroid received the lowest absorbed doses.

The mean effective dose (ED) was 0.0167 mSv/MBq for females and 0.0157 mSv/MBq for males (range: 0.0114–0.0207 mSv/MBq).

## 4. Discussion

[^18^F]F-AraG, a radiolabeled AraG analog, was developed as an agent for imaging activated T cells and is currently being investigated as a biomarker of response to immunotherapies. Here, we report the results of a pilot study that evaluated biodistribution, dosimetry, and safety of [^18^F]F-AraG in six healthy participants. Administration of [^18^F]F-AraG was found to be well tolerated and safe. No adverse effects to the study participants were noted.

In general, the observed biodistribution of [^18^F]F-AraG was reflective of the expression (Figure 3(c)) [26] and, more importantly, activity profiles of the enzymes involved in its accumulation in cells, dGK, and SAMHD1. Deoxyguanosine kinase is constitutively expressed and shows low tissue specificity [27]. The activity of the dGK enzyme, the critical determinant of [^18^F]F-AraG's cellular trapping, was assessed in the bovine tissues and showed to be the highest in the liver and lowest in the brain, with intermediate levels in the heart and thymus [28]. While the liver and heart showed significant [^18^F]F-AraG uptake, a low signal was detected in the thymus and the brain. The low thymus uptake, most likely resulting from involuted thymus in adult population, was also observed for radiolabeled nucleoside analogs targeting deoxycytidine kinase, a kinase closely related to dGK and with increased expression in lymphoid tissue [29].

Although the low signal in the brain may be suggestive of F-AraG's inability to cross intact blood-brain-barrier (BBB), the recent preclinical findings [25] as well as nucleoside utilization in the brain and other findings do not support this notion. Nucleoside transporters, found on the brain blood vessels and at the BBB [30], allow entry of nucleosides synthesized *de novo* in the liver [31] into the brain parenchyma, while the complex enzymatic machinery of the salvage pathway fine-tunes the homeostasis of nucleoside pools needed for proper brain function. The functional interaction between dGK and SAMHD1 in the salvage pathway seems to be of particularly high significance for regulation of dGTP levels in the brain. The brain, along with the liver, is the organ most affected by the mtDNA depletion caused by dGK deficiency [11]. SAMHD1 activity exacerbates mtDNA depletion and pathology associated with it [19]. SAMHD1 deficiency also affects the brain, resulting in an inflammatory neurodegenerative disorder, Aicardi–Goutiéres syndrome (AGS). Considering the importance of the balanced dNTP pools, it is plausible that the signal in the brain reflects low physiological accumulation of deoxyguanosine in the brain achieved by the activity of SAMHD1, found to be highly expressed in the brain. Other tissues with high expression of SAMHD1, such as the muscle and bone marrow, also show low [^18^F]F-AraG uptake. In the liver and kidneys, tissues with the highest [^18^F]F-AraG accumulation, SAMHD1 protein has not been detected [32]. As dGK and SAMHD1 activity are directly linked with mitochondria, on a higher level, we expected [^18^F]F-AraG to exhibit a similar biodistribution pattern as imaging agents that accumulate in mitochondria, such as 99 m Tc-MIBI. Indeed, [^18^F]F-AraG's accumulation in the heart, thyroid, and salivary glands closely resembled the one of 99 m Tc-MIBI in those organs [33].

In addition to the mitochondrial biogenesis in the tissue regulated by the functional interplay between dGK and SAMHD1, the signal observed in various organs may also reflect the presence and functional status of the T cells transiting or residing there. Tissue-resident T cells (T_RM_) are a special subset of long-lived effector memory T cells that do not circulate in the blood and allow rapid response upon antigen re-encounter [34]. Tissue-resident CD8^+^ T cells have been described across different tissues and are particularly important in barrier tissues where they can provide immediate local immunity against environmental pathogens. Furthermore, tumor infiltrating T_RM_s express check point inhibitor receptors such as PD-1 and were found to be crucial for antitumor immunity [35] and associated with survival in melanoma patients [36]. The presence and role of T_RM_ in the organs that showed high [^18^F]F-AraG uptake, such as the salivary glands [37, 38], liver [39, 40], and kidneys [41], have been well established. Interestingly, the liver, previously considered only in the context of immunotolerance, is now actively investigated as a lymphoid organ [42] that can affect nonhepatic immune responses [43] and immunotherapy outcome [44]. Considering these findings, it seems prudent to investigate whether the [^18^F]F-AraG uptake in the liver and other T_RM_-rich organs could provide information useful for assessing systemic response to T cell-modulating therapies.

The estimated ED for [^18^F]F-AraG was 0.0162 mSv/MBq (0.0167 mSv/MBq for females and 0.0157 mSv/MBq for males), similar to estimates for other nucleoside tracers such as FLT (0.0305 mSv/MBq) [45] and [18F]CFA (0.0203 mSv/MBq) [46], as well as the most commonly used tracer FDG (0.0199 mSv/MBq) [47]. A dose of 185 MBq will result in 3.1 mSv exposure to the patient, a value comparable to 6-7 mSv exposure for a typical FDG scan and well below 30 mSv limit in human research set by the Radioactive Drug Research Committee (RDRC). The kidneys received the highest absorbed dose (critical organ) while radiation-sensitive organs, such as red marrow, testes, and ovaries, were well within RDRC defined limit.

The limitations of this study include a small number of participants and data points in time-activity curves. Although the number of participants is typical for this type of study, the relatively small sample size may not be representative of the physiological accumulation in a larger population. Future studies will involve a larger cohort of subjects and continuous imaging that will allow a comprehensive evaluation of baseline values.

## 5. Conclusion

Evaluation of [^18^F]F-AraG in healthy human subjects showed its suitability for clinical imaging. [^18^F]F-AraG was well tolerated, with a radiation dosimetry profile similar to other commonly used [^18^F]-labeled tracers. Its biodistribution was reflective of [^18^F]F-AraG's proposed mechanism of intracellular accumulation. [^18^F]F-AraG's association with mitochondrial biogenesis and favorable biodistribution characteristics make it an attractive tracer with a variety of potential applications.

## Figures and Tables

**Table 1:** Patients' demographic data.

Participant no.	Sex	Age (y)	Height (cm)	Weight (kg)	Injected dose (MBq)	Effective dose (*μ*Sv/MBq)
1	M	60	167	75.7	299.7	20.7
2	M	39	167	63	321.9	11.4
3	F	55	152	46.7	325.6	18.9
4	M	24	156	46.4	244.2	15.1
5	F	30	164	92.1	329.3	14.7
6	F	60	163	55.8	321.9	16.4
Mean ± SD		44.7 ± 15.8	161.5 ± 6.1	63.3 ± 17.9	307.1 ± 32.5	16.2 ± 3.3

**Table 2:** Imaging time points.

Participant	30 min scan start (min)			
	Scan 1	Scan 2	Scan 3	Scan 4
1	0	47	102	143
2	0	56	111	153
3	0	47	89	134
4	0	39	90	131
5	0	40	88	104
6	0	44	94	139

**Table 3:** Estimated radiation dose for [^18^F]F-AraG in humans.

Mean absorbed dose (mGy/MBq)/equivalent dose (mSv/MBq)		
Organ	Adult female (58 kg)	Adult male (73.7 kg)
Adrenals	0.0181	0.0126
Brain	0.0020	0.0017
Breasts	0.0020	0.0016
Gallbladder Wall	0.0170	0.0139
LLI Wall	0.0033	0.0230
Small intestine	0.0231	0.0127
Stomach Wall	0.0069	0.0050
ULI Wall	0.0090	0.0061
Heart Wall	0.0262	0.0142
Kidneys	0.3063	0.2370
Liver	0.0721	0.0570
Lungs	0.0090	0.0074
Muscle	0.0033	0.0025
Ovaries	0.0046	
Pancreas	0.0135	0.0101
Red marrow	0.0046	0.0037
Osteogenic cells	0.0029	0.0020
Skin	0.0016	0.0012
Spleen	0.0442	0.0253
Testes		0.0008
Thymus	0.0021	0.0015
Thyroid	0.0004	0.0004
Urinary bladder wall	0.0239	0.0232
Uterus	0.0047	
Total body	0.0068	0.0051
Effective dose (mSv/MBq)	0.0167	0.0157

## Data Availability

The data included in the study are available from the corresponding author upon reasonable request.
